# Diversity in 113 cowpea [*Vigna unguiculata* (L) Walp] accessions assessed with 458 SNP markers

**DOI:** 10.1186/2193-1801-3-541

**Published:** 2014-09-20

**Authors:** Kenneth F Egbadzor, Kwadwo Ofori, Martin Yeboah, Lawrence M Aboagye, Michael O Opoku-Agyeman, Eric Y Danquah, Samuel K Offei

**Affiliations:** West Africa Centre for Crop Improvement, University of Ghana, Legon, Accra Ghana; CSIR – Plant Genetic Resources Research Institute, Bunso, Ghana; Cocoa Research Institute of Ghana, Tafo, Ghana; The Biotech Centre – University of Ghana, Legon, Accra Ghana; Department of Crop Science, University of Ghana, Legon, Accra Ghana

**Keywords:** Characterization, Cowpea, Diversity, Genetic, Genotype, Markers, SNPs

## Abstract

Single Nucleotide Polymorphism (SNP) markers were used in characterization of 113 cowpea accessions comprising of 108 from Ghana and 5 from abroad. Leaf tissues from plants cultivated at the University of Ghana were genotyped at KBioscience in the United Kingdom. Data was generated for 477 SNPs, out of which 458 revealed polymorphism. The results were used to analyze genetic dissimilarity among the accessions using Darwin 5 software. The markers discriminated among all of the cowpea accessions and the dissimilarity values which ranged from 0.006 to 0.63 were used for factorial plot. Unexpected high levels of heterozygosity were observed on some of the accessions. Accessions known to be closely related clustered together in a dendrogram drawn with WPGMA method. A maximum length sub-tree which comprised of 48 core accessions was constructed. The software package structure was used to separate accessions into three groups, and the programme correctly identified varieties that were known hybrids. The hybrids were those accessions with numerous heterozygous loci. The structure plot showed closely related accessions with similar genome patterns. The SNP markers were more efficient in discriminating among the cowpea germplasm than morphological, seed protein polymorphism and simple sequence repeat studies reported earlier on the same collection.

**Electronic supplementary material:**

The online version of this article (doi:10.1186/2193-1801-3-541) contains supplementary material, which is available to authorized users.

## Introduction

Cowpea [*Vigna unguiculata* (L) Walp] is an important staple food crop in Ghana and many other parts of the world (Obembe 2008; Timko and Singh 2008). The crop is also used as animal feed. As a legume, cowpea fixes nitrogen and therefore, contributes to soil improvement. Compared with other important staples such as maize, rice, yams and plantains in Ghana, most cowpea varieties have shorter maturity period (55 days for some varieties) making it a crop of choice to address hunger and malnutrition.

Cowpea is known to be relatively drought tolerant (Boukar *et al.*^2011^; Muchero *et al.*^2009^) and this attribute results in its cultivation mainly in the savanna and forest – savanna transitional zones of West Africa. Resource inputs in cowpea production are relatively low compared to those used in the production of other major staples, making its cultivation affordable by resource poor farmers (Muchero *et al.*^2009^).

Cowpea is primarily a self-pollinating crop and its genetic base is considered to be narrow (Sharawy and El-Fiky 2002; Fang *et al.*^2005^; Asare *et al.*^2010^). Presence of diversity in the germplasm of crops is essential for successful crop improvement (Varshney *et al.*^2007^). Limited genetic diversity poses a threat to the survival of a species as this limits ability to respond to changes in climate, pathogen populations and agricultural practices (Manifesto *et al.*^2001^). The source of genetic resources for crop improvement is the available germplasm in genebanks and this need to be assessed for availability of useful traits for crop improvement (Tan *et al.*^2012^).

Cowpea is one of the most researched crops at the genebank of the Council for Scientific and Industrial Research – Plant Genetic Resources Research Institute (CSIR – PGRRI) (Bennett-Lartey 1992; Asare *et al*. 2010). CSIR – PGRRI is situated at Bunso, in the Eastern Region of Ghana. Most of these germplasm were collected in the 1980s and 90s from different parts of Ghana. These have been characterized based on morphological (Bennett-Lartey 1992) seed protein (Oppong-Konadu *et al.*^2005^) and Simple Sequence Repeat (SSR) differences (Asare *et al.*^2010^).

Single Nucleotide Polymorphism markers (SNPs) are powerful tools in genetic diversity study in living organisms (Deulvot *et al*. 2010). SNPs are more effective in diversity assessment compared with other markers such as AFLPs and SSRs (Varshney *et al*. 2007). Using morphological markers, Cobbinah *et al.* (2011) observed multiple duplicates within cowpea germplasm in Ghana. Reason for the high number of duplicates was the limited number of morphological markers and the low genetic variability these markers revealed. Asare *et al.* (2010) using SSRs could also not discriminate between some accessions of the cowpea germplasm in reference. It is critical, for the purposes of efficiency, that the best available tool for genetic diversity assessment is deployed.

SNPs are numerous in the genome of plants and other living organisms (Galeano *et al.*^2009^; Deulvot *et al.*^2010^) and they serve as good tools for diversity studies (Acquaah 2007; Varshney *et al.*^2007^). SNPs may be the best choice for diversity studies at the moment. As of 2012, there had been no report on cowpea diversity studies that used SNPs markers (Tan *et al.*^2012^). However, in 2013, Huynh *et al.* (2013) and Lucas *et al.* (2013b) reported their diversity work on worldwide cowpea collection. The objectives of this study were to use SNP markers to:

1. Assess genetic diversity within cowpea germplasm assembled from CSIR – PGRRI, Bunso, Ghana and abroad.
2. Use diversity information to select a core cowpea germplasm collection for breeding purposes.
3. Help guide future international research in cowpea breeding.

## Materials and methods

### Plant materials

**Figure 1:**
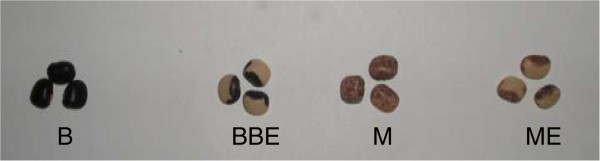
**Different lines of Gh4524 based on seed coat colour differences: B, BBE, M and ME for black, big black eye, mottle and mottle eye respectively.**

A total of 113 cowpea accessions were characterized. These included 102 accessions collected from different parts of Ghana. One hundred and one accessions of the 102 are being conserved at CSIR – PGRRI genebank at Bunso, Ghana, while one accession (WACCI01) was obtained from West Africa Centre for Crop Improvement (WACCI), University of Ghana. Four accessions were breeding lines selected from accession GH4524 based on seed coat colour differences (Figure 1). Six of the accessions were improved varieties in cultivation in Ghana, namely: ‘Asontem’, ‘Nhyira’, ‘Zaayura’, ‘Tona’, ‘Paddy Twua’ and ‘Bawuta’. In addition there were two lines each from University of California Riverside (UCR779 and CB27) and International Institute of Tropical Agriculture (IITA) in Nigeria (IT97K-556-6 and IT82E-18). The accession labeled “market” is one of the popular cowpea imported to Ghana from Togo and was, therefore, included in the imported accessions. All the accessions are listed in Table 1.

**Table 1:** **Passport data of the cowpea accessions used for the experiment**

GH number	Local name	Collection date	Region	Lat	Long	Seed colour	Structure ID	% Hetero
GH1622	Ayi-dze	21-12-82	Volta	06°35'N	00°27'E	Black	81	0.43
GH1630*	Asedua	01-12-83	Volta	06°29'N	00°10'E	Black	59	0.71
GH1665*	Asedua	26-11-82	Eastern			Cream	80	31.45
GH1667	AduaNsawa	30-11-82	Eastern	06°40'N	01°20'W	Brown	29	0.21
GH2279	Sanji	14-10-87	North	09°15'N	00°37'W	Red	96	0.67
GH2280*	Sanji	14-10-87	North	09°15'N	02°10'W	Brown	85	0.43
GH2281*	Sanji	14-10-87	North			Dark	95	0.24
GH2282*	Sanji	14-10-87	North	09°55''N	00°07'W	Black	84	0.00
GH2284*	Sanji	14-10-87	North	09°18'N	02°25'W	Red	107	6.99
GH2288*	Isagi	14-10-87	North	09°26'N	02°00'W	Black	41	0.85
GH2291*	Sanji	15-10-87	North	09°13'N	01°02'W	Red	68	3.81
GH2293	Sanji	15-10-87	North	09°29'N	01°13'W	Red	74	0.22
GH2294*		15-10-87	North	09°29'N	01°13'W	Mottled	102	0.51
GH2296	Sanji	15-10-87	North	10°21'N	00°35'W	Red	53	0.21
GH2306	Bonda	16-10-87	Upper West	10°40'N	02°01'W	Black	101	0.21
GH2307	Bondawa	16-10-87	Upper West	10°53'N	02°07'W	Black	92	0.21
GH2309*	Bibitakone	17-10-87	Upper West	10°50'N	03°15'W	Black	25	7.86
GH2312	Dapiala	17-10-87	Upper West	10°15'N	02°27'W	Red	17	0.45
GH2314*	Bengah	17-10-87	Upper West	10°0'N	02°24'W	Mottled	76	2.50
GH2316*	Bengah	17-10-87	Upper West	09°26'N	02°30'W	Mottled	106	0.23
GH2317	Achibe	17-10-87	North	09°32'N	02°36'W	Mottled	90	0.21
GH2323*	Bengah	19-10-87	North	08°25'N	02°17'W	White	88	1.49
GH2325	Asedua	30-11-87	Eastern	06°22'N	00°24'W	Cream/Mixed	91	0.43
GH2326	Asedua	30-11-87	Eastern	06°22'N	00°24'W	White	46	0.21
GH2328	Asedua	30-11-87	Eastern	06°22'N	00°24'W	Red	86	0.22
GH2329	Asedua	30-11-87	Eastern	06°22'N	00°24'W	White	55	0.21
GH2330	Asedua	30-11-87	Eastern	06°22'N	00°24'W	Red	27	0.64
GH2331	Asedua	30-11-87	Eastern	06°22'N	00°24'W	Mottled	54	0.42
**GH2332**	Asedua	30-11-87	Eastern	06°22'N	00°24'W	White	47	0.21
GH2333	AduaNsawa	30-11-87	Eastern	06°22'N	00°35'W	Mottled	112	0.85
GH2334*	AduaNsawa	30-11-87	Eastern	06°22'N	00°35'W	Cream	103	0.21
GH2335	AduaNsawa	12-1-87	Eastern	06°22'N	00°35'W	Red	100	0.21
GH2340	AduaNsawa	12-1-87	Eastern	06°34'N	00°35'W	Black	42	0.00
GH2341	AduaNsawa	12-1-87	Eastern	06°38'N	00°34'W	White	24	0.21
GH2342*	AduaNsawa	12-1-87	Eastern	06°38'N	00°34'W	Red	83	0.63
GH2347*	Yor	18-12-87	Eastern			Black	97	0.00
GH2492*	-	20-10-87	Ashanti			White	62	1.35
GH3667*	Ayi	23-10-93	Volta	06°18'E	0°11'N	Dark	89	0.21
GH3668	Ayi	23-10-93	Volta	06°18'E	0°11'N	Black	43	0.43
GH3669	Ayi	23-10-93	Volta	06°18'E	0°11'N	Black	28	0.21
Gh3670*	Ayi	23-10-93	Volta	06°35'N	0°06'E	Brown	94	0.43
Gh3673	Ayi	24-10-93	Volta	06°41'N	0°17'E	Black	69	0.64
Gh3674*	Eveyi	24-10-93	Volta	06^0^34'N	0°18'	Black	77	0.63
Gh3675	Ase fita	25-10-93	Eastern	06°13'N	0°5'E	Black	26	0.64
Gh3677*	Yor	25-10-93	Eastern	06°06'N	0°12'W	Brown	99	0.43
Gh3685	Asedua	30-10-93	Eastern	07°16'N	02°19'W	Black	108	0.43
Gh3689	Sanji	1-11-93	Eastern	08°54'N	0°39'N	Brown	34	0.43
GH3701	Sanji	11-2-93	Eastern	06°10'w	0°03'N	Brown	93	0.42
Gh3706*	Benga	4-11-93	North	09°50'N	0°29'W	Brown	87	0.00
GH3703	Tua	11-2-93	North			Red	50	0.47
GH3708*	Sega	11-4-93	North	09°20'W	02°20'N	Mottled	105	0.45
GH3710*	Tua	11-5-93	North			Dark	109	0.21
Gh4028	Adua nsawa	19-5-96	North			Red	57	0.23
GH4524	Yor	29-7-96	Accra			Black	1,2,3,4	0.24
GH4529	Tolonye	30-7-96	Accra			Mottled	65	0.43
GH4530	Yor	30-7-96	Accra			Brown	18	0.43
GH4532	Ayiyibor	31-7-96	Volta			Red	61	0.65
GH4533*	Ayi	31-7-96	Volta			Cream	82	1.32
GH4537*	Ayi	08-1-96	Volta			Brown	60	0.22
GH4541	Ayi	08-1-96	Volta			Mttled	52	0.63
GH4546	Ayi	08-3-96	Volta			Dark	70	0.21
GH4769*	Bianga	22-10-96	Upper West			Red	63	0.42
GH4771	Bianga	22-10-96	Upper West			Black	38	0.21
GH4778	Gonja	27-10-96	North			Red	23	0.21
GH5038	Vakli	11-4-96	Volta			Cream	33	0.21
GH5039	Ekye	11-4-96	Eastern			Red	66	0.44
GH5040*	Yor	11-5-96	Eastern			Red	40	0.21
GH5044	Yor	11-6-96	Eastern			Red	39	0.21
GH5045	Yor	11-6-96	Eastern			Red	44	0.66
GH5048	AduaNsadua	11-6-96	Eastern			Red	37	0.21
GH5049	Asedua	11-6-96	Eastern			Red	72	0.21
GH5050	Asedua	11-8-96	Ashanti			Red	32	0.42
GH5344	Asedua	11-6-96	Ashanti			Red	48	0.42
Gh5346	Asedua	9-11-96	Ashanti			Brown	51	0.64
GH6045*	Soronko		Ashanti			Reddish Brown	30	0.63
GH6060*	Ayiyi	28-8-98	Eastern			Cream	22	0.21
GH7167	Tue	18-2-03	Upper East			White	104	0.42
GH7174	Tse	18-2-03	Upper East			Cream	49	0.42
GH7178*	Benga	19-2-03	Upper East			Red	21	36.49
Gh7185	Sona	19-2-03	Upper East			Black	71	0.21
GH7187	Goatana	20-2-03	Upper East			Black	13	0.21
Gh7218	Sona	04-1-03	Upper West			Dark	58	0.00
GH7222	Bondabene	04-1-03	Upper West			Brown	56	0.66
GH7224	Sompla	04-1-03	Upper West			Dark	31	0.21
GH7226*	Bene	04-1-03	Upper West			White	111	0.43
GH7228	Bene	04-2-03	Upper West			Dark	45	3.24
GH7229	Bene	04-2-03	Upper West			Dark	19	0.21
GH7230*	Bene	04-2-03	Upper West			Brown	79	0.24
GH7231	Bene	04-2-03	Upper West			Dark	67	2.44
GH7233	Bene	04-2-03	Upper West			Brown	35	0.42
GH7234*	Bene	04-2-03	Upper West			Dark	110	20.09
GH7235	Bene	04-2-03	Upper West			Dark	75	0.21
GH7243		04-2-03	Upper West			Dark	78	0.42
Gh7245*	Sonorni	1-4-03	Upper West			Brown	98	0.43
Gh7273		1-4-03	Upper West			White	73	0.63
GH7875	Asedua	15-3-06	Eastern			Red	20	0.21
GH7888*	Asetenapa	16-8-06	Ashanti			Cream	36	0.63
IT97K556			IITA, Nigeria			Brown	5	0.43
IT82E-18*			IITA, Nigeria			Brown	6	0.88
UCR 779*			UCR, USA			Brown	7	0.21
CB 27*			UCR, USA			White	8	0.64
Bawuta*			CSIR – SARI			White	14	0.21
Tona*			CSIR – CRI			Brown	9	0.42
Nhyira*			CSIR – CRI			Cream	10	0.42
PaddyT*			CSRI – SARI			White	15	0.21
Asontem*			CSIR – CRI			Red	16	2.77
Market*			Volta (Ho)			White	64	2.70
WACCI01*			Legon, Accra			Dark	11	0.66
Zayuraa*			CSIR – SARI			White	12	1.07

Note: *Lat* Latitude, *Long* Longitude, *Hetero* Heterozygosity. * - Accessions selected as core for further studies.

Seeds were germinated in sterilized top soil contained in nursery boxes at the Crop Science Department Garden, University of Ghana. Leaf discs of one week old plants were sampled from one plant per accession and shipped to the laboratory of KBiosciences in the United Kingdom where genomic DNA was extracted. The DNA samples were genotyped using 500 SNPs from the cowpea panel (Muchero *et al*. 2009; Lucas *et al*. 2011).

### Markers used

**Figure 2:**
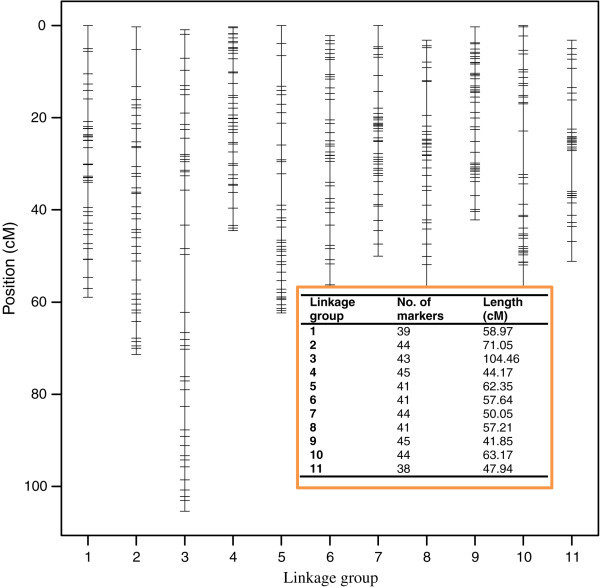
**The SNP markers used for the experiment – Length of linkage group, number of markers and their positions.**

The SNP markers used were distributed across the cowpea genome. Figure 2 shows a map of the eleven linkage groups of cowpea and indicate the positions of the markers on the cowpea genome. The length of each linkage group and their respective number of markers are inserted. Twelve out of the 477 SNP markers were unmapped, thus summing up to 465 instead of 477 markers (Figure 2).

### Data analysis

The software Darwin (Perrier and Jacquemoud-Collet 2006) was used to analyze the data. Dissimilarity was calculated using simple matching coefficient after Perrier *et al*. (2003) as follows:


Where:

*dij*: dissimilarity between units*i* and *j*

*L*: number of loci

π: ploidy

*ml*: number of matching alleles for locus *l* (Perrier *et al*. 2003).

The calculated dissimilarity coefficient was used to construct a tree using the hierarchical clustering of Weighted Paired Group Method with Arithmetic Mean (WPGMA). It was used for a factorial plot and a Maximum Length sub-tree was constructed to select a representative core accessions.

Detection of the underlying genetic population among the studied cowpea accessions was carried out with the Structure software (Pritchard *et al*. 2000). Three populations (K = 3) were assumed and indicated with blue, green and red colours. Different numbers were tried for K and finally 3 accepted with admixture ancestry model. Length of burnin was 5000 and the number of MCMC was set at 10000.

## Results

### Allelic diversity

Out of the 477 SNPs, 458 were polymorphic. SNP data revealed that some of the markers although polymorphic, only few, sometimes just one genotype had them in the collection. The percentage of the cowpea accessions that shared common allele per locus, thus, varied greatly: from 0% versus 100% to 50% versus 50% (refer to Additional file 1).

### Heterozygosity

Some of the cowpea accessions were heterozygous at some of the marker loci. Heterozygosity at a locus may indicate accessions undergoing segregation. Many of the accessions in the collection had at least one heterozygous site (Table 1, column 9).

### Factorial plot of the cowpea accessions

**Figure 3:**
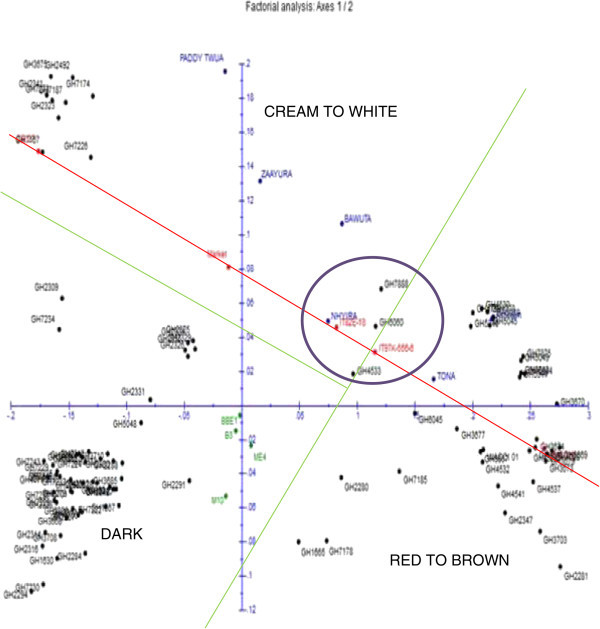
**Factorial display of 113 cowpea accessions.**

General diversity of the germplasm is displayed in a factorial plot in Figure 3 and Additional file 2. Lines and a circle were drawn in Figure 3 to aid in explanation. Three major clusters were identified in the Figure demarcated by the “y” shaped two green lines. Members of each cluster were characterized mostly by similar seed coat colour.

### Dendrogram of the cowpea accessions

**Figure 4:**
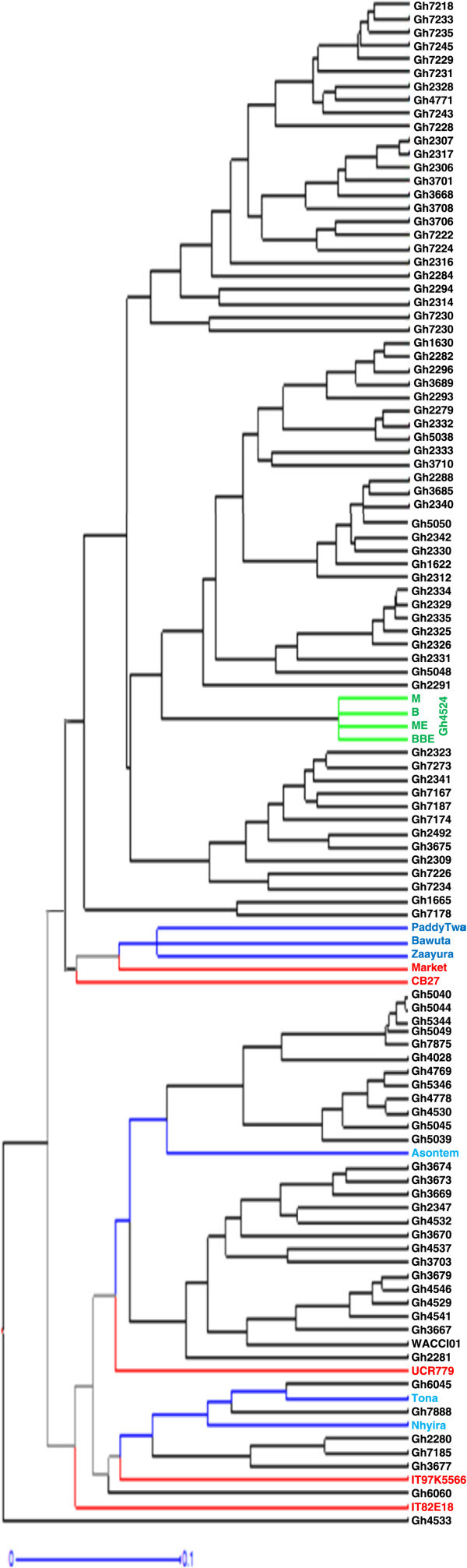
**Dendrogram of 113 cowpea accessions constructed from dissimilarity using 458 polymorphic SNP markers.**

**Figure 5:**
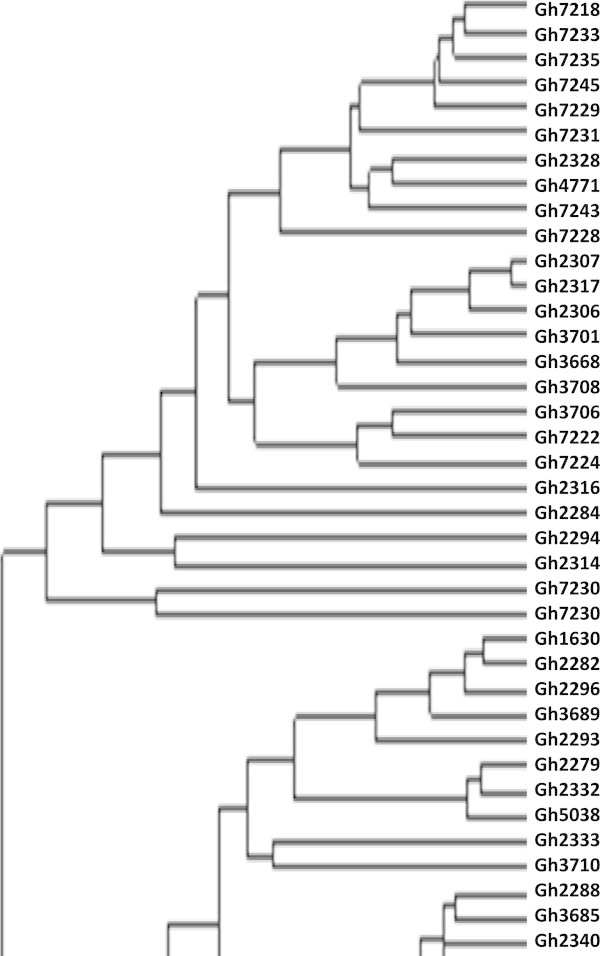
**First 38 cowpea accessions in Figure** 4 **.**

**Figure 6:**
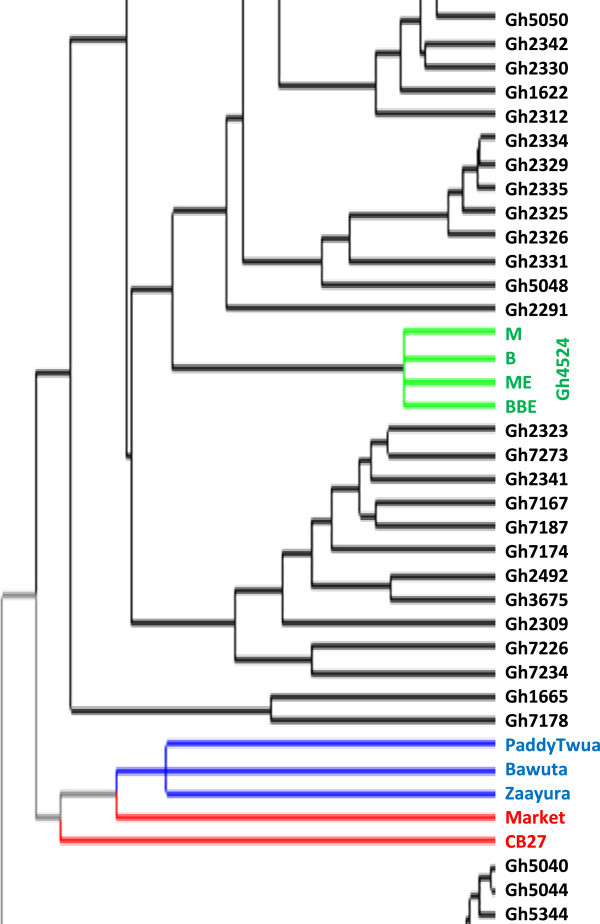
**Middle 38 cowpea accessions in Figure** 4 **.**

**Figure 7:**
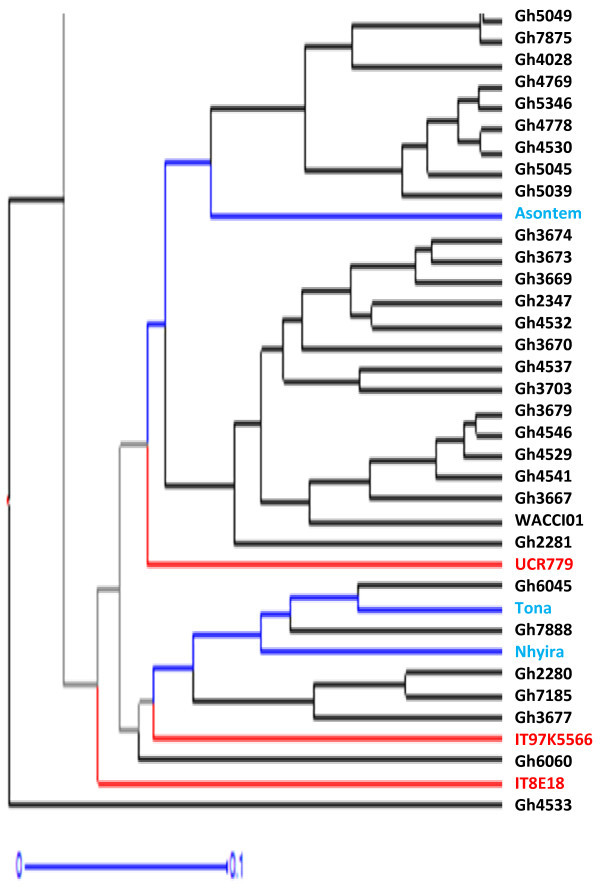
**Final 37 accessions of Figure** 4 **.**

Figure 4 is a dendrogram of the cowpea accessions drawn with the calculated values of dissimilarity using hierarchical clustering with WPGMA method. Accessions written in black represent genebank materials obtained from Bunso and WACCI01 while those in blue and green are improved varieties from Ghana and Gh4524 lines respectively. Accessions in red are from IITA, UCR and the one named “market”. For legibility purposes, different portions of Figure 4 were shown in Figures 5, 6 and 7.

### Result of structure analysis

**Figure 8:**
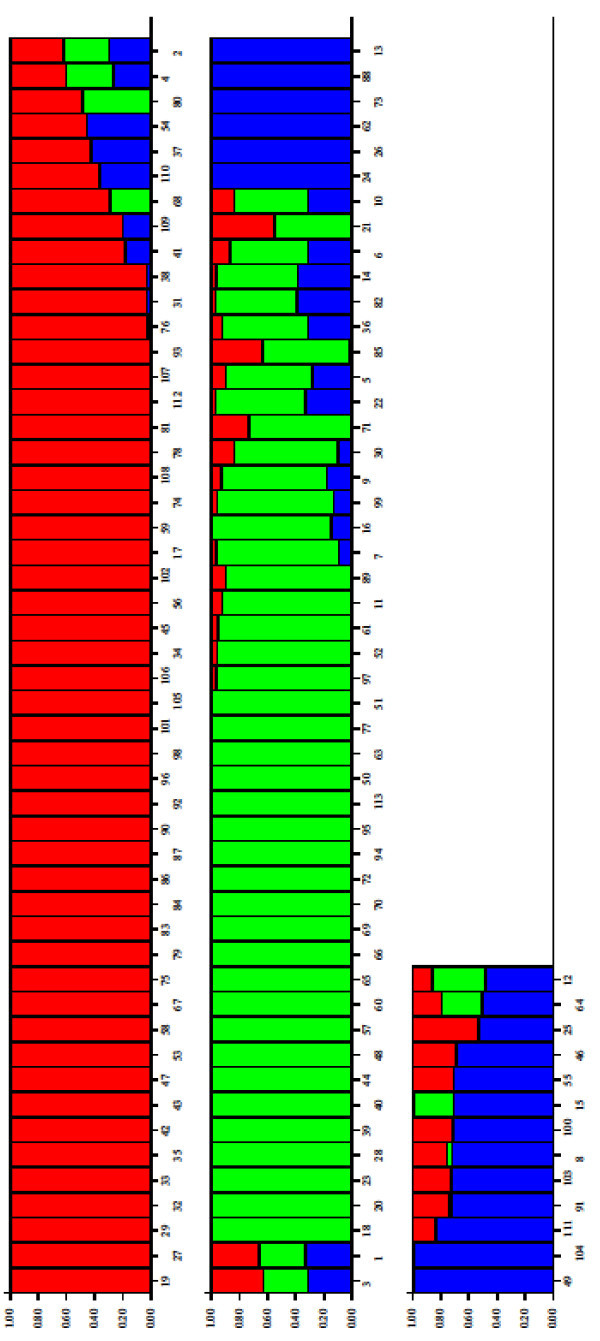
**Estimated population structure for the cowpea accessions studied.**

The result of analysis made with Structure is presented in Figure 8. Each accession is represented by a vertical line, which is partitioned into coloured segments indicating the estimated membership fractions for it. Estimated Ln Prob. of data = -32014.2: Mean value of ln likelihood = 701.5: Mean value of alpha = 0.071: Mean value of Fst 1 = 0.7: Mean value of Fst 2 = 0.56: Mean value of Fst 3 = 0.49. Accessions in the same population in Figure 8 were collected from across the different agro-ecological zones of Ghana with no known history of genetic relationship between most of them.

### Core 48 accessions

**Figure 9:**
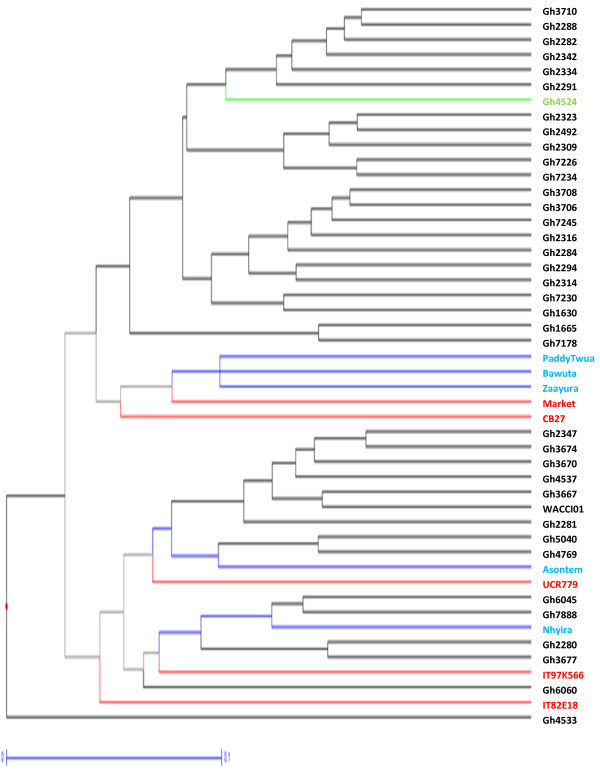
**Dendrogram of 48 core cowpea accessions identified through Maximum Length Sub Tree method for conservation and breeding purposes.**

Maximum length sub tree method (Perrier *et al*. 2003) was used to identify forty-eight core accessions for breeding purposes (Figure 9). Accessions in red represent foreign materials; black for genebank materials, blue for improved varieties and green for Gh4524 line. These 48 accessions are very diverse morphologically. The core 48 accessions include UCR779, CB27, IT97K-556-6 and IT82E-18 which are internationally known cowpea lines. These materials have unique alleles that are not likely to be available in the genebank of PGRRI. Five of the improved varieties from Ghana such as ‘Asontem’ and ‘Nhyira’ are also in the core 48.

## Discussion

For purposes of discussion, we denote those alleles present in not more than 10% of the studied collection as ‘rare alleles’. The cowpea accessions, GH7888 (a genebank material), ‘Zaayura’ and IT97K-556-6 shared a rare allele. IT97K-556-6 is IITA line while ‘Zaayura’ is a commercial variety released by CSIR - Savanna Agricultural Research Institute. Another rare allele was shared by UCR779 and ‘Zaayura’. UCR 779, a Botswana landrace, resistant to aphid (Muchero *et al*. 2009) was one of the most unique accessions in the collection. It was the only line with “A” against “T” for one marker. “Asontem” (IT82E-16) which is one of the improved varieties in Ghana developed by IITA in collaboration with CSIR-Crops Research Institute, also had a rare allele at a locus. The last example of a rare allele observed in the collection was “T” for GH7167, GH2288 and CB 27 where all other accessions had “C”. The allelic diversity thus varied greatly for the studied cowpea accessions.

The 458 SNP markers were able to discriminate between all the cowpea accessions studied. Previous studies (Bennett-Lartey 1992; Oppong-Konadu *et al.*^2005^; Asare *et al.*^2010^) could not discriminate all accessions, but the increase number of markers used here and their high levels of polymorphism allowed the discrimination of even closely related accessions such as BBE, M, B and ME (segregated lines of Gh4524). This confirms the robustness of SNP markers in diversity studies as reported by Varshney *et al.* (2007). The separation of the four Gh4524 lines on the other hand could most probably be due to segregation as Lucas *et al*. (2013a) were able to identify duplicates from the USDA’ core cowpea collection with SNP markers.

Definite patterns were identified in the cowpea collection. Both Ghanaian and foreign elite accessions clustered together (Figures 3 and 4). Patterns could also be seen in Figure 4 based on the seed coat colour similarities of the cowpea accessions. However, accessions collected from different regions of Ghana did not cluster together in most cases. Asare *et al.* (2010) also did not observe strong geographic relationship in the PGRRI cowpea collection when they used SSR in diversity studies. Tanhuanpaa and Manninen (2012) in their studies on *Phleum pretense* with SSRs also did not observe significant correlation between the various accessions and their geographic origins. Geography does not always reflect underlying genetic structure (Rosenberg *et al*. 2002).

Only 150 markers which are about 30% did not have any cowpea showing heterozygosity. Some markers generally revealed higher levels of heterozygosity. There were 23 accessions heterozygous for a particular marker. Most of these accessions clustered together (Figures 3 and 4). Gh7234 for instance had as many as 90 heterozygous sites. This suggests that some of the genebank cowpea accessions are not pure. Phenotypic analysis strengthened the assertion that seeds of Gh7234 were different in terms of seed coat colours with the dominant as dark mottling. Similar observation was made for Gh7231 which had 13 heterozygous sites. However, some of the improved varieties including the foreign ones (IT97K-556 and CB27) also had one or more heterozygous sites. High heterozygosity known in such crops as plantains (Tenkouano *et al*. 1999), Scot pines (Gupta *et al*. 2001) and cassava (Dyer *et al*. 2011) was unanticipated in this study. The high heterozygosity observed in some of the cowpea accessions might be due to outcrossing (Lucas *et al*. 2011; Kouam *et al*. 2012) during regeneration at the genebank and to the fact that some of them have hybrid origin. There were only five accessions (Gh2282, Gh2340, Gh2347, Gh3706 and Gh7218) that were homozygote for all the loci.

Three major clusters are identified on the factorial display of the accessions indicated by two green lines which formed a “y” shape (Figure 3). Accessions of same cluster generally have similar seed coat colours with only few exceptions. Some of these exceptions are Gh2281 and Gh7185 with dark seed coat colours clustering in the red to brown seed coat colour group while Gh2284 and Gh5048 with red seeds clustered with dark colours. Seed coat colour is a frequently used as a morphological trait in classifying crop varieties (Adesoye and Ojobo 2012) and may also be linked with other important traits (Atis *et al*. 2011). The clusters according to the seed coat colours are; Dark, Cream to White and Brown to Red (Figure 3). The boundaries between the dark seed coat colour cluster and the other two were very conspicuous. However, the boundary between the white and red seed coat colour clusters was not very clear. Six accessions in the purple outlined circle formed a sub-cluster between the white and red seed coat colour clusters. Even though, the six accessions formed a sub-cluster, each individual was closely linked to its respective major cluster, with the exception of IT82E-18 (Figure 3). In contrast, Asare *et al.* (2010) did not observe clustering pattern based on seed coat colour when they characterized cowpea collection with SSRs. However, in this study clear pattern based on seed coat colour was observed. Similarly in maize, SNP markers were used to identify kernel colour gene (Sharma *et al*. 2011).

All the foreign accessions fell on a straight line (red). Meaningfully, they also fell in their appropriate colour seed coat clusters. These are elite germplasm (improved varieties) and have been selected for similar traits over a long period of time. The improved varieties from both Ghana and abroad are found on or above the red line (Figure 3). Local accessions that clustered with these elite accessions could be very useful materials for cowpea breeding programmes, especially in Ghana, for being genetically close to the elite varieties and being adapted to the local climate. For instance, the dissimilarity between GH7888 (a genebank material), and ‘Zaayura’ was as small as 0.026. GH7167, GH2288 and CB 27 clustered together. CB27 was released in California in 1999 and is resistant to *Fusarium* wilt race 3 and moderately susceptible to aphid (Muchero *et al.*^2009^). Phenotypically CB27 did not share much similarity with Gh2288. Seed mass of CB27 was twice that of Gh2288. The kidney shaped seed of CB27 had white seed coat with black eye. This type of cowpea has a high preference in Ghanaian markets (Langyintuo, 2003). Gh2288 on the other hand had dark mottling seed coat colour. Accession CB27 was erect while Gh2288 was prostrate. Few traits shared by CB27 and Gh2288 are, pigmented immature pod tip which dry up to straw, pendant pods and sub-hastate terminal leaflet that were slightly curved.

No elite genotype fell in the dark coat coloured cluster (Figure 3). Commercial varieties of cowpea are mainly white or brown to red coat coloured in Ghana as they are the types preferred by consumers (Quaye *et al.*^2011^; Langyintuo *et al.*^2003^). Separation of many Ghanaian accessions away from elite and commercial varieties may mean availability of diversity that could be exploited for cowpea improvement. Despite claims of limited genetic variation in cowpea (Asare *et al*. 2010; Kumar *et al*. 2011 Tan *et al.*^2012^), there is substantial morphological and genetic evidence that cowpea is a very diverse taxon (Huynh *et al.*^2013^). This experiment has shown that the studied germplasm has some amount of diversity that can be used for cowpea improvement. Furthermore the cowpea community should consider the many subspecies of cowpea and the tens of thousands of accessions collected from more than 50 countries that are available through different germplasm collections. Special interest would be to use the landraces in broadening the genetic base of the improved cowpea varieties similar to what was suggested for asparagus bean in China (Tan *et al*. 2012).

Clustering of materials such as CB27, Paddy Twua (Padi Tuya), ‘Bawuta’ and ‘Zaayura’ is very significant. This is because Padi ‘Tuya’ and a number of varieties released by CSIR – SARI are known to have parentage from California Black eye (Padi *et al.*^2004^). Close relationship between “Market” and CB27 (Figures 3 and 4) was also not surprising. “Market” was an imported cowpea picked from a market and was suspected to originate from California Black-eye because of its seed features. Clustering of CB27 and Market had confirmed their relatedness. The dendrograms in Figures 6 and 7 support pedigree knowledge as seen in the clustering of Gh4524 lines and UCR779 with IT82E-18 which are both from South/East Africa, Botswana and Mozambique, respectively. The SNP markers were for that matter very reliable in this diversity study. An exception was that IT82E-18 did not cluster with Asontem (IT82E-18 in Ghana). It could probably be that the Asontem collected was not the IT82E-18 as it has been in the hands of farmers for a long time. Farmers might be calling a morphological similar variety Asontem. Another possibility resulting in the non-clustering of IT82E-18 and the supposed Asontem is that the plant genotyped as Asontem could be a rogue as described by Luca *et al.* (2013b).

Three populations were assumed and represented by different colours; blue, green and red with 8, 22 and 38 accessions discretely coming from them respectively (Figure 8). Thus the total number of accessions without admixed genome was 68. Members in the blue population, some of which are Gh2323, Gh7167 and Gh7174 clustered at the top left corner in Figure 3. In the exception of Gh7178 (13 in Figure 5), all the accessions with entirely blue genome have white or cream seed coat colour. Gh2323 and Gh7273 which are both members of the blue population in Figure 8 were the closest relatives in Figure 4. The cowpeas in the green population in Figure 8 are mostly red seed coated and also showed close relationship in the dendrogram in Figure 4. Accessions such as Gh5039, Gh5040 and Gh5049 in the green population clustered together in Figure 3. Similar patterns were also observed for the red population in Figure 8. However, accessions in this group are more diverse in terms of seed coat colour. The clustering pattern in the dendrogram and factorial plot with “Darwin” thus had some similarities with that of “Structure”. Some authors believe that the software Structure does not always create clusters that are consistent with evolutionary history of individuals in populations; however, it is one of the most frequently used software for cluster analysis (Kalinowski 2011). In this study the result of the structure analysis made biological sense especially when it is compared to the phenotype of the cowpea accessions and the analysis made with Darwin.

Different combinations of admixture genome for different cowpea accessions were observed. Some of the accessions had genome from two different populations while others were from all the three. All of the improved varieties had genome from different populations (Figure 5). “Zaayura”, “CB27” and “Market” had similar patterns in the exception of having slightly different proportions for the various segments. These three varieties are believed to have been bred from materials with common parentage (Padi *et al*. 2004). The four accessions obtained from Gh4524 (numbers 1, 2, 3 and 4 in Figure 8) showed very similar patterns and had portions of their genome from different sources. Some other accessions from the genebank as well showed inheritance of genome from different populations. Cowpea is predominantly inbreeding and it is shown by the mean apha value of 0.07 indicating that most of the accessions are essentially from one population. However, mean value of 3.4% outcrossing has been reported (Kouam *et al*. 2012) which might be the reason for some of the genebank materials to be admixed. The observation in this study thus confirms this phenomenon.

The establishment of a core germplasm collection helps in easy management and identification of variations for breeding purposes (van Hintum *et al*. 2000). Where the germplasm collection is very large, management goes beyond core to mini core collection (Upadhyaya *et al*. 2010). Forty-eight core accessions were consequently, identified from the fingerprinting for conservation and crop improvement. The core 48 accessions include UCR779, CB27, IT97K-556-6 and IT82E-18 which are internationally known cowpea lines. These materials had unique alleles that are not likely to be available in the genebank in Ghana. Five of the improved varieties from Ghana were included in the 48 core accessions. These 48 accessions include all of the 11 improved varieties in the study. Bringing these improved accessions which were hitherto not in the collection into the activities of the genebank might mean expansion of the gene pool of the cowpea which is considered to be narrow (Tan *et al*. 2012). Expansion of gene pool is important for crop improvement (Varshney *et al.*^2007^).

The sphericity index as explained by Perrier *et al.* (2003) was considered in choosing the 48 core cowpea accessions. The sphericity index for all the 113 accessions was 0.69. This figure meant that there was much redundancy in the collection, compared to the final three accessions which had the highest sphericity index of 1. The core 48 accessions selected had sphericity index of 0.79 which was quite low indicating much redundancy which could permit further reduction in the number of accessions included in the core. However, as much as 10 improved varieties were included in the core when the number of accessions was reduced to 20 with sphericity index of 0.88. This meant that with 20 core accessions, only 50% would be from the genebank. To avoid further narrowing of the genetic base of the cowpea germplasm for breeding purposes (Sharawy and El-Fiky 2002; Fang *et al*. 2005; Asare *et al*. 2010; Tan *et al.*^2012^), the 48 core accessions were, therefore, accepted to increase the genetic base of the core.

The core accessions varied in morphological traits such as growth habit where there were a wide range spanning from erect to spreading types. Plant pigmentation, leaf shape and flower colour also varied among the core accessions. Seeds with different coat colours, sizes and shapes were found within the core accessions. Some of the accessions in the core collection had been reported to have resistance to biotic stresses. Examples include CB27 and UCR779 which are resistant to *Fusarium* wilt and aphid respectively (Muchero *et al*. 2009). These accessions could be used as parents to develop varieties resistant to biotic stresses such as aphid borne mosaic virus which is a serious constraint to cowpea cultivation in many parts of Africa (Orawu *et al*. 2012). Further evaluation of the core 48 accessions may reveal other traits that might be of interest to cowpea breeders.

## Conclusion

This study was one of the earliest diversity studies in cowpea using SNP markers. The markers were efficient in discriminating between all the accessions used in the study including closely related materials such as Gh4524 lines. Accessions known to be related by ancestry such as CB27 and Paddy Twua, clustered together, demonstrating the reliability of the markers. The information provided in this diversity study could be useful in cowpea improvement in Ghana and elsewhere. A total of 48 accessions were identified as a core collection for breeding purposes. These core accessions were morphologically very diverse and included UCR779, CB27, IT97K-556-6 and IT82E-18. These are elite materials obtained from different countries. Improved varieties from Ghana such as ‘Asontem’ and ‘Nhyira’ are also in the 48 core accessions. The genetic diversity of the selected core could be of importance for future plant breeding for the development of superior varieties of cowpea.

## Electronic supplementary material

Additional file 1: **Raw SNP Genotype Data used for Analyses.** (XLSX 435 KB)

Additional file 2: **Factorial plot of the cowpea accessions.** (DOCX 48 KB)
