# Supplementary figures and images for: Diversity in 113 cowpea [Vigna unguiculata (L) Walp] accessions assessed with 458 SNP markers
Source: Springerplus. 2014 Sep 20;3:541. doi: 10.1186/2193-1801-3-541 (PMC4190189; doi:10.1186/2193-1801-3-541)

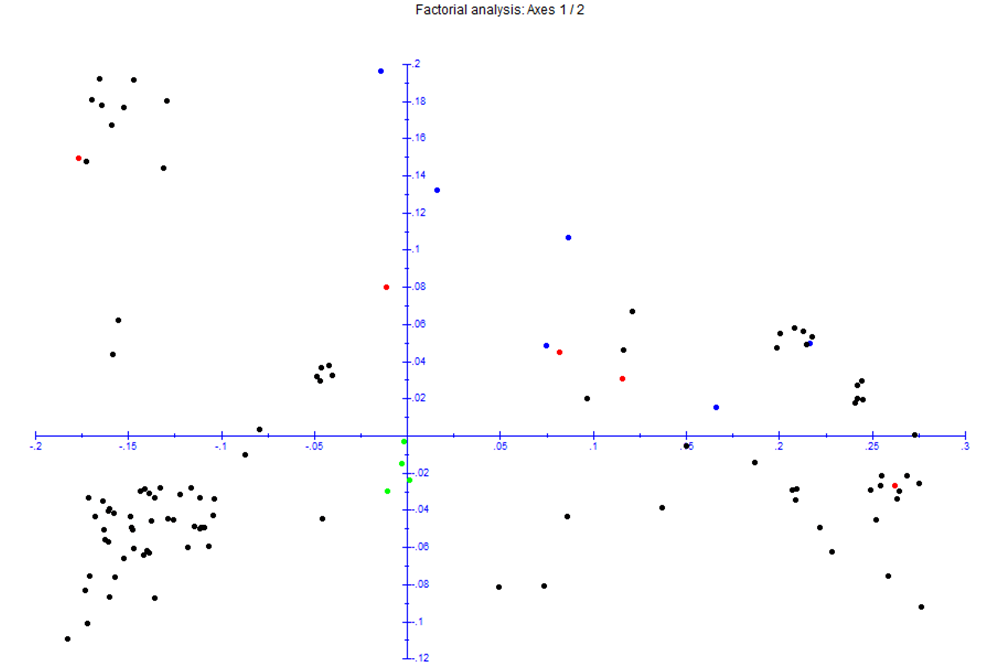


Factorial plot of the cowpea accessions.

Supplement: Supplementary file 2 — Additional file 2: Factorial plot of the cowpea accessions. (DOCX 48 KB) [file 40064_2014_1267_MOESM2_ESM.docx]
